# Composition and Structure of NiCoFeCr and NiCoFeCrMn High-Entropy Alloys Irradiated by Helium Ions

**DOI:** 10.3390/ma16103695

**Published:** 2023-05-12

**Authors:** Bauyrzhan Amanzhulov, Igor Ivanov, Vladimir Uglov, Sergey Zlotski, Azamat Ryskulov, Alisher Kurakhmedov, Mikhail Koloberdin, Maxim Zdorovets

**Affiliations:** 1 Institute of Nuclear Physics, Almaty 050032, Kazakhstan; igor.ivanov.inp@gmail.com (I.I.); ryskulov_nbd@mail.ru (A.R.); kurahmedov.alisher@gmail.com (A.K.); koloberdin@inp.kz (M.K.); mzdorovets@inp.kz (M.Z.); 2 Department of Nuclear Physics, New Materials and Technologies, Physical-Technical Faculty, L.N. Gumilyov Eurasian National University, Astana 010008, Kazakhstan; 3 Engineering Profile Laboratory, L.N. Gumilyov Eurasian National University, Astana 010008, Kazakhstan; 4 Department of Solid State Physics, Belarusian State University, 220030 Minsk, Belarus; uglov@bsu.by (V.U.); zlotski@bsu.by (S.Z.)

**Keywords:** high-entropy alloys, radiation resistance, microstress, X-ray diffraction, Rutherford backscattering

## Abstract

High-entropy alloys (HEAs) have prospects for use as nuclear structural materials. Helium irradiation can form bubbles deteriorating the structure of structural materials. The structure and composition of NiCoFeCr and NiCoFeCrMn HEAs formed by arc melting and irradiated with low-energy 40 keV He^2+^ ions and a fluence of 2 × 10^17^ cm^−2^ have been studied. Helium irradiation of two HEAs does not change the elemental and phase composition, and does not erode the surface. Irradiation of NiCoFeCr and NiCoFeCrMn with a fluence of 5 × 10^16^ cm^−2^ forms compressive stresses (−90 … −160 MPa) and the stresses grow over −650 MPa as fluence increases to 2 × 10^17^ cm^−2^. Compressive microstresses grow up to 2.7 GPa at a fluence of 5 × 10^16^ cm^−2^, and up to 6.8 GPa at 2 × 10^17^ cm^−2^. The dislocation density rises by a factor of 5–12 for a fluence of 5 × 10^16^ cm^−2^, and by 30–60 for a fluence of 2 × 10^17^ cm^−2^. Stresses and dislocation density in the HEAs change the most in the region of the maximal damage dose. NiCoFeCrMn has higher macro- and microstresses, dislocation density, and a larger increase in their values, with an increasing helium ion fluence compared to NiCoFeCr. NiCoFeCrMn a showed higher radiation resistance compared to NiCoFeCr.

## 1. Introduction

Modern challenges facing the scientific and technological part of nuclear power are associated, to a large extent, with increasing the efficiency of nuclear power plants by increasing their operating temperatures [1,2,3]. Materials for the core and protection of reactors must withstand temperatures of up to 500–850 °C [4,5,6]. Moreover, such reactors will experience high-dose neutron irradiation. Structural materials must withstand radiation loads of up to 100 dpa (displacements per atom) [7], and according to other sources, up to 400–600 dpa (equivalent to 80 years of reactor operation) [4]. Classical materials, such as austenitic steels, nickel and other metal alloys, with a base of one or two elements, undergo phase transformations and a decomposition of solid solutions at elevated temperatures, corrode upon contact with liquid coolants and many gases, and swell at high doses of neutron irradiation [2,3]. In addition, in such conditions, they are prone to embrittlement and the loss of operational properties. Austenitic and ferritic–martensitic steels resist degradation at temperatures of up to 400–500 °C; however, at higher temperatures, long operation for more than a year, and increased mechanical stress, tensile strength decrease, grain growth and precipitation of second phases occur [8]. To solve these problems, it is necessary to develop new materials, since the current structural materials of nuclear plants are not capable of long-term operation in chemically aggressive environments at elevated temperatures. Achieving this goal will also give impetus to the development of jet propulsion systems and elements of aerospace vehicles [1,2,3,9].

One of the most promising classes of materials for solving such problems are high-entropy alloys (HEA) [1,2,3,9,10,11,12]. They attract the attention of scientists from all over the world, and the works of the Cantor, Senkov, Yeh teams are recognized as pioneers in this field [13,14,15].

HEA is defined as an alloy of usually five or more basic elements in equimolar ratios, where the concentration of each element is in the range of 5–35 at.% [15]. HEAs have a structure that is different from most known homogeneous alloys: it cannot always be represented as a solid solution lattice based on the lattice of one element due to the high degree of disorder in this structure [1,12]. The increased entropy of mixing elements contributes to minimizing the value of the Gibbs free energy and, thus, increasing the thermodynamic stability of the HEA. Taking into account the variety of types of solid solutions that can be created, theoretically, HEA-type materials can have absolutely any properties [16,17]. HEAs based on Ni, Fe, Cr and Mn were found to have high corrosion resistance [15,18], while ceramic phase additions, such as TiC, can further raise the corrosion resistance of HEA-based coatings [19]. Nickel-based HEAs have a stable strength and hardness at high temperatures [15], and their wear resistance and strength can be further increased with ceramics such as WC [20].

It is also worth noting the presence of the so-called “recovery effect” in HEA, which is mentioned by the authors of many works on the radiation resistance of these alloys [21,22]. This effect implies the possibility of returning the lattice to its original state (before irradiation) in samples irradiated with ions by neutralizing radiation-induced defects. By selecting the correct value of the operating temperature, for example, it is possible to achieve a permanent restoration of the HEA structure during irradiation using the “self-healing effect” directly in the process of operation [21,22,23,24,25,26]. In the NiCoFeCrMn alloy, the migration energies of interstitial atoms and vacancies differ little or even intersect [27,28], which accelerates recombination and increases the possibility of defect annihilation, reducing the number of defects.

Nickel HEAs with an FCC structure, namely, alloys based on NiCoFeCr and an alloy of NiCoFeCrMn, showed high resistance to radiation defects when irradiated with helium. It was found that when irradiated with helium ions, helium bubbles are formed in the FeCoNiCr HEA, as in steels, but their density number is higher and their size is smaller than in pure nickel or austenitic steels in the temperature range of 523–973 K [29]. When irradiated with helium ions, the size of helium bubbles increases with temperature in the HEA based on Ni–Co–Fe–Cr, and the density number of these bubbles decreases [29,30,31]. In addition, it was found that the density of helium bubbles in CoCrFeMnNi increased with the fluence of helium ions during irradiation, but decreased with increasing temperature, and reached 5 × 10^23^ m^−3^ at the highest fluence of 2 × 10^16^ cm^−2^ [32].

Studies have shown that helium diffusion, and, accordingly, the formation of bubbles, passes from a mechanism based on the replacement of interstitial atoms to a vacancy mechanism with an increase in temperature [29,30]. Furthermore, at relatively low temperatures, the diffusion of helium in HEA, Ni and austenitic steel occurs according to a similar mechanism of replacement of its own interstitial atoms and helium, but the proportion of helium diffusing into bubbles in HEA is less than in Ni, which can be caused by lattice distortion and disordering of the alloy [30]. In HEA, lattice distortion and stress fields affect the formation of point defects, and thus affect the energies of migration, defect formation and the helium diffusion coefficient itself [29]. The formation of helium bubbles and blisters in Ni and Ni-based HEAs under the same irradiation conditions and at different fluences needs to be studied to improve the understanding of the He-associated defect formation.

When irradiated with helium and heavy ions, as well as during annealing, the segregation of HEA elements is possible. Irradiation-induced grain boundary segregation leads to a decrease in the Mn concentration and an increase in the Ni/Co concentrations [27]. One of the reasons for segregation is the asymmetric diffusion of one element and ordering [33]. When annealed at high temperatures of up to 1073 K and 1173 K, a Cantor’s NiCoFeCrMn alloy retains its FCC solid solution structure for up to 500 days [29,34]. However, in CrMnFeCoNi, upon prolonged annealing of 500 days at a temperature of 773 K, three phases with a high concentration of Cr, Ni and Mn, Fe, and Co are formed [34], and at 973 K, precipitates with a high concentration of Cr with a sigma-phase structure emerge [34,35]. Therefore, the elemental distribution stability under irradiation and different temperatures needs to be studied.

All of the above makes HEAs promising materials for use in nuclear power plants and aircraft power plants [22,25,26,36,37].

HEAs can be divided into three main groups: based on 3D transition metals, refractory metals and rare earth elements. The most economically justified and, therefore, the most frequently considered are the first two groups of alloys [38]. Nickel-containing HEAs with an FCC structure show increased radiation resistance in comparison with steels and nickel supermetals of the inconel type [11]. At the same time, the reasons for this behavior of nickel-containing HEAs still do not have an exact explanation or description, which is facilitated by conflicting data on their properties and probable operational capabilities.

Crystal distortion is caused by the microstrain, which is stimulated by the stresses and defect formation under irradiation. Most similar research was concerned with microstrain and lattice parameter changes in HEAs. In HEAs and steels including 316L and 304H, helium irradiation usually leads to lattice expansion [22,39]. Helium irradiation led to lattice expansion in VCrFeMn [39]. However, in HEAs such as non-equiatomic Ti_2_ZrHfV_0.5_Mo_0.2_, lattice parameters can be reduced by helium ion irradiation due to higher lattice distortion [22]. Lattice expansion and microstrain can also be increased by irradiation with non-heavy ions such as Ni and C. Irradiation by Ni ions resulted in lattice expansion in FeCoNiCr and FeCoNiCrMn [40]. Irradiation of (WTiVNbTa)C_5_ high-entropy ceramics by carbon ions at higher fluences led to higher microstrain and lattice expansion at room temperature, while higher temperature irradiation led to a lower microstrain [41]. However, the irradiation of cold-worked SS316L by heavy energetic ions such as Ne^6+^ showed that microstrain decreased with ion fluence increase due to dislocations present before the irradiation, since interstitials annihilated at dislocations and recombined with vacancies [42]. Moreover, microstrains decreased when steel with smaller grains was irradiated by neutrons to 1.37 dpa compared to larger-grain steel [43]. Therefore, the novelty of this research lays in the analysis of stresses, structure and composition of HEAs, both in the maximal radiation damage and maximal implantation regions at a high irradiation fluence.

The purpose of this work was to study and compare the radiation resistance of the HEAs of CoCrFeNi and CoCrFeMnNi, as well as to reveal the mechanisms of defect behavior upon irradiation with helium ions.

## 2. Materials and Methods

**Figure 1:**
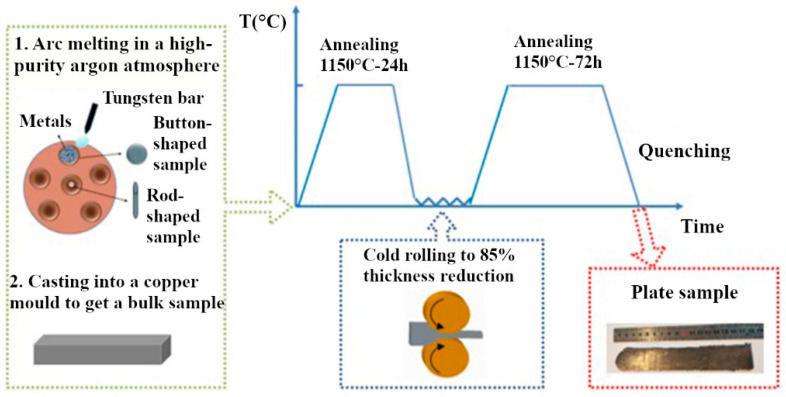
High-entropy sample preparation scheme.

CoCrFeNi and CoCrFeMnNi alloys and the reference Ni sample were obtained from the Beijing Institute of Technology (Beijing, China) by the following technology. Bulk ingots were prepared from powders of pure (up to 99.97%) metals by arc-melting in a high-purity argon atmosphere, followed by casting into copper cuvettes. After their crystallization, annealing was carried out for 24 h at 1150 °C, in order to spheroidize and homogenize the grain structure of the samples (Figure 1). Subsequently, cold-rolling was carried out until the thickness of the ingots decreased by 85%, and the final annealing at 1150 °С for 72 h was carried out in order to reduce the texture and stresses caused by rolling.

All samples had the shape of rectangular parallelepipeds with linear dimensions of 5.0 mm × 5.0 mm × 1.5 mm.

The samples were irradiated at a DC-60 heavy ion accelerator at the Astana branch of the Institute of Nuclear Physics (Astana, Kazakhstan). Irradiation was carried out with He^2+^ ions with an energy of 40 keV at fluences of 5 × 10^16^ and 2 × 10^17^ cm^−2^. This type of ion emerges in reactors as a result of the interaction of neutrons with atoms of matter, followed by alpha decay, which leads to the formation of gas bubbles, areas of increased internal stresses, accumulation of helium in the material and, as a result, to its swelling.

The study of the elemental composition and depth distribution of elements in the samples of FCC-structured high-entropy CoCrFeNi and CoCrFeMnNi alloys was carried out on a DC-60 cyclotron using a combination of Rutherford backscattering of heavy ions (heavy-ion Rutherford backscattering spectrometry, HIRBS) and particle-induced X-ray emission (particle-induced X-ray emission, PIXE), [44] the main advantages of which are rapidity, non-destructive analysis, high resolution of elements in terms of mass and depth. For HIRBS/PIXE measurements, a collimated beam of ^14^N^2+^ nitrogen ions with an energy of 14 MeV was used. The sample under study was mounted on a high-precision (0.01°) triaxial goniometer in a vacuum chamber, which made it possible to precisely control the orientation of the sample relative to the nitrogen ion beam. The backscattered particles were recorded by an Au–Si semiconductor surface-barrier detector. The registration angle was 160°, the energy resolution of the detector was ~11 keV. The spectra of the backscattered particles were processed using RUMP (Rutherford universal manipulation program) [45]. RUMP is specially designed to process the backscattered particle spectra accumulated in the analyzer, making it possible to simulate experimental RBS spectra, calculate the layer-by-layer elemental composition of samples and build element distribution profiles in the sample. The concentration profile of elements in RUMP is represented in the form of a successive alternation of layers of varying thickness and composition, the concentration of elements which is specified in the form of a stoichiometric formula.

The surface morphology of the samples was analyzed via scanning electron microscopy (SEM) on a ZEISS LEO 1455 VP scanning electron microscope and the images were obtained at an accelerating voltage of 20 kV.

The phase analysis of the samples was carried out by X-ray phase analysis. The X-ray patterns were obtained on a Rigaku Ultima IV X-ray diffractometer in parallel beam geometry, using CuKα characteristic X-ray radiation with a wavelength of λ = 0.154179 nm. The X-ray diffraction patterns were taken with a constant rotation of the samples at a speed of 30 rps to eliminate the influence of the texture of the alloys. Images of the samples were taken in the small-angle X-ray diffraction (SAXRD) mode at an X-ray beam incidence angle α to study only the irradiated area of the samples. Internal stresses in the samples were determined by the g-sin^2^ψ method [46]. This method makes it possible to determine stresses in near-surface layers by changing the angle of incidence of X-ray radiation α (penetration depth). The calculation of microstresses and dislocation density was carried out using the Williamson–Hall method [47]. This method makes it possible to isolate the contribution of microstresses and sizes of coherent scattering regions (CSRs) to the broadening of diffraction peaks. The dislocation density (ρ) was calculated using the formula ρ = 3/D^2^, where D is the CSR size.

## 3. Results

### 3.1. Composition and Structure of Unirradiated HEAs CoCrFeNi and CoCrFeMnNi

**Figure 2:**
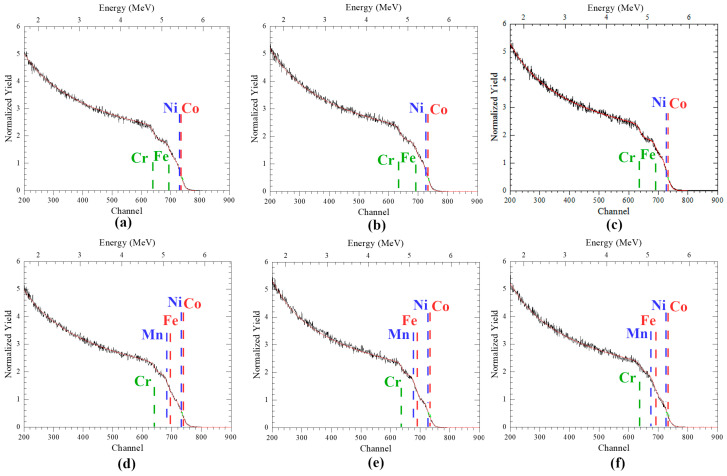
HIRBS spectra of: (**a**,**d**) unirradiated and irradiated by He^2+^ ions (40 keV) with fluences of (**b**,**e**) 5 × 10^16^ cm^−2^ and (**c**,**f**) 2 × 10^17^ cm^−2^, (**a**–**c**) CoCrFeNi and (**d**–**f**) CoCrFeMnNi HEAs.

The results of the study of the elemental composition by the HIRBS/PIXE method of the unirradiated CoCrFeNi and CoCrFeMnNi alloys are shown in Table 1 and Figure 2. As can be seen in Table 1, the compositions of the CoCrFeNi and CoCrFeMnNi alloys are close to equiatomic, at about 25 and 20 at.%, respectively. Small deviations in the concentrations of Ni and Co, as well as Fe and Mn, are associated with close masses of these elements, which makes it impossible to accurately determine the concentration of each element separately.

Figure 2 shows the results of the analysis of the elemental composition and distribution of the elements over the depth of the NiCoFeCr and NiCoFeCrMn HEA samples by the HIRBS method during simulation using the RUMP program, in which the theoretical spectrum (red line) is fitted to the experimentally obtained spectrum (black line). As can be seen in Figure 2, the theoretical and experimental spectra completely coincide, which makes it possible to determine the elemental composition and distribution of elements over the depth of HEA samples with high accuracy (measurement error no more than 6%), the data of which are presented in Table 1.

The analysis of spectra, obtained using HIRBS methods, showed that unirradiated alloys have a homogeneous distribution of elements by depth (Figure 2a–c).

**Figure 3:**
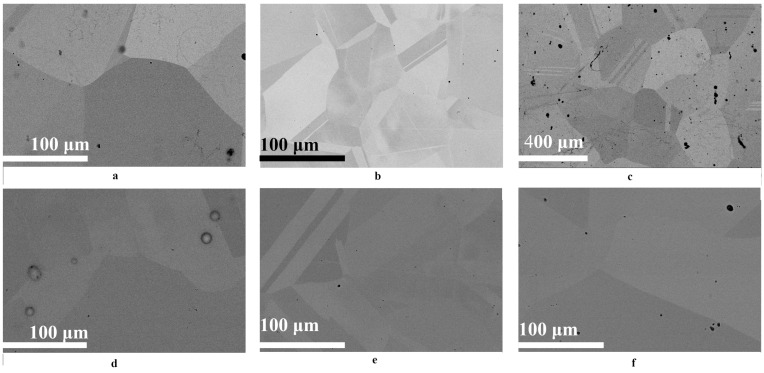
SEM images of the surfaces of (**a**–**c**) unirradiated and (**d**–**f**) irradiated (by He^2+^ ions (2 × 10^17^ cm^−2^)) (**a**,**d**) Ni, and (**b**,**e**) the NiCoFeCr HEA and (**c**,**f**) NiCoFeCrMn HEA samples. SEM images taken approximately at (**a,b,d**–**f**) 1000× and (**c**) 200× magnification.

The results of studying the structure of the original CoCrFeNi and CoCrFeMnNi alloys and the Ni sample using the SEM method are shown in Figure 3. It can be seen that the samples have a coarse-grained structure, with a grain size of about 200 μm for Ni, 80 μm for CoCrFeNi and 100 μm for CoCrFeMnNi (Figure 3a–c). In all samples, the grains have a regular polygonal shape with traces of twinning introduced by cold-rolling.

**Figure 4:**
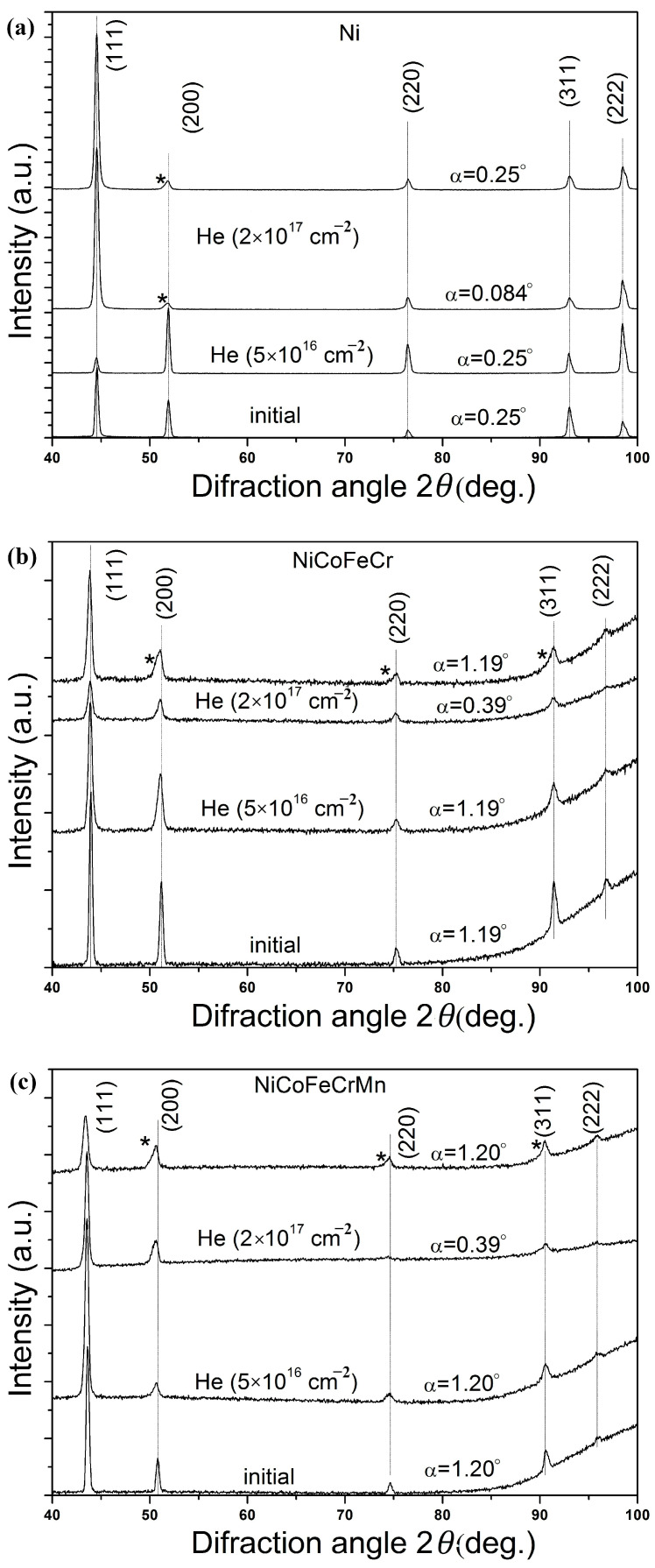
XRD patterns of unirradiated and irradiated (by He^2+^ ions (2 × 10^17^ cm^−2^)) (**a**) Ni, (**b**) NiCoFeCr and (**c**) NiCoFeCrMn HEA samples, obtained at different angles of incidence α of X-ray irradiation. The asterisk denotes the shoulder of asymmetric diffraction peaks.

The results of studying the phase composition of the initial samples are shown in Figure 4. The X-ray patterns were obtained at a small angle of incidence of X-ray radiation α = 0.084, 0.25, 0.39, 1.19 and 1.20° for Ni, CoCrFeNi and CoCrFeMnNi, respectively. Angles α = 0.084, 0.39° correspond to the X-ray penetration depth of 77 nm, and α = 0.25, 1.19 and 1.20° to a depth of 231 nm.

The analysis of the formed phases and comparison of the angular positions of their diffraction peaks with the peaks of pure FCC metals included in the composition of the alloys showed that the HEA peaks are located between the corresponding peaks of pure metals, which indicates the formation of a single-phase system in the form of substitutional solid solutions (Ni, Co, Fe, Cr) and (Ni, Co, Fe, Cr, Mn) with an FCC lattice. The nickel samples represent the Ni phase with the FCC lattice. The lattice constant calculated from the X-ray diffraction patterns for the CoCrFeNi alloy was 0.35716 ± 0.00009 nm, for the CoCrFeMnNi alloy, it was 0.35987 ± 0.00009 nm and for nickel, it was 0.35217 ± 0.00003 nm.

**Figure 5:**
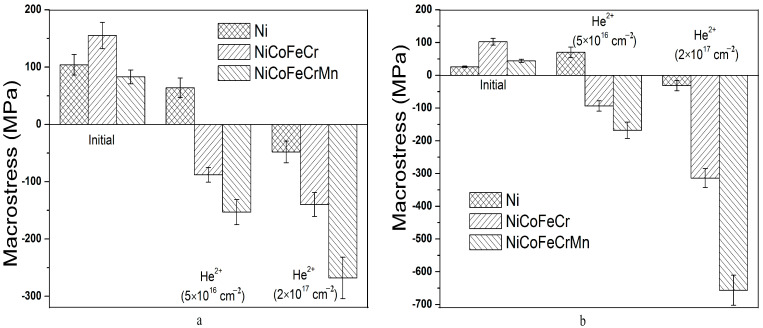
Macrostresses in unirradiated and irradiated (by He^2+^ (40 keV) ions) Ni, and NiCoFeCr and NiCoFeCrMn HEA samples at Region 1 (**a**) and Region 2 (**b**).

**Figure 6:**
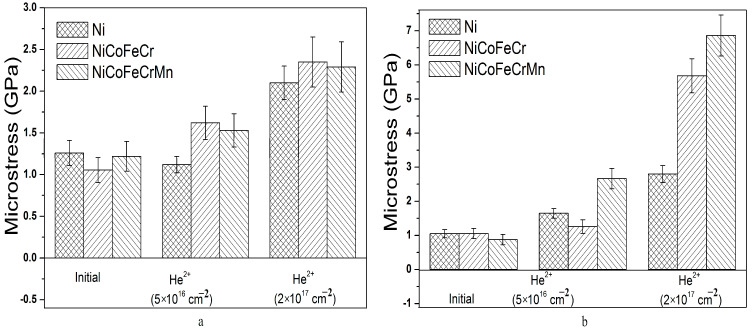
Microstresses in unirradiated and irradiated (by He^2+^ (40 keV) ions) Ni, and NiCoFeCr and NiCoFeCrMn HEA samples at Region 1 (**a**) and Region 2 (**b**).

**Figure 7:**
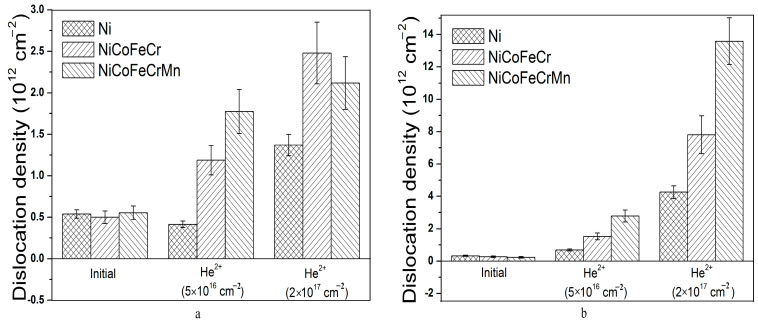
Dislocation density in unirradiated and irradiated (by He^2+^ (40 keV) ions) Ni, and NiCoFeCr and NiCoFeCrMn HEA samples at Region 1 (**a**) and Region 2 (**b**).

It was found that tensile stresses formed in Ni, and NiCoFeCr and NiCoFeCrMn alloys amounted to 26 ± 2, 103 ± 10 and 44 ± 5 MPa, respectively (Figure 5). The microstresses and dislocation density calculated using the Williamson-Hall method were 1.05 ± 0.12 GPa and (0.33 ± 0.04) × 10^12^ cm^−2^, 1.05 ± 0.15 GPa and (0.26 ± 0.05) × 10^12^ cm^−2^, 0.88 ± 0.15 GPa and (0.234 ± 0.05) × 10^12^ cm^−2^ for Ni, and NiCoFeCr and NiCoFeCrMn alloys, respectively (Figure 6 and Figure 7). The formation of tensile stresses is associated with cold-rolling in the process of HEA formation.

### 3.2. Composition and Structure of CoCrFeNi and CoCrFeMnNi HEAs Irradiated by Helium Ions

The radiation resistance of the composition and structure of the HEA CoCrFeNi and CoCrFeMnNi was studied under irradiation with low-energy He^2+^ ions, with an energy of 40 keV and fluences of 5 × 10^16^ and 2 × 10^17^ cm^−2^. Preliminary calculations of radiation damage and implanted helium concentration were carried out using the stopping and range of ions in matter program (SRIM-2013) [48], using the quick Kinchin–Pease mode. The measured sample densities of 8.908, 8.144 [33] and 7.964 [49] g/cm^3^ for Ni, NiCoFeCr and NiCoFeCrMn, respectively, were used, along with an assumed threshold displacement energy of 40 eV for all elements [50].

**Figure 8:**
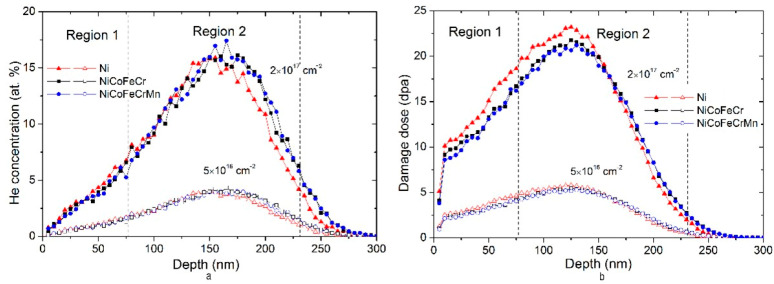
Distribution of (**a**) implanted helium and (**b**) damaging dose in Ni, NiCoFeCr and NiCoFeCrMn HEAs irradiated by He^2+^ (40 keV) ions.

Figure 8 shows the distribution profiles of the implanted helium and the damaging dose in Ni, and CoCrFeNi and CoCrFeMnNi alloys irradiated with He^2+^ ions (40 keV). SRIM calculations showed that the projective range of helium ions in the samples is 146 nm, and the maximal energy loss in the region of up to 100 nm is 0.22 keV/nm.

As can be seen in Figure 8, the maximal concentrations of the implanted helium and the damaging dose are 4.3 at.% and 5.7 dpa, 16 at.% and 23 dpa for fluences of 5 × 10^16^ and 2 × 10^17^ cm^−2^, respectively. The distributions of the implanted helium and the damaging dose are normal with asymmetry near the surface, which is typical for implanted profiles at ion energies above 10 keV. This is due to the fact that at the beginning of the range, helium ions have a high energy, and the role of nuclear deceleration is small. The ions are scattered through small angles and their trajectories are more straightforward.

Studies of the elemental composition of NiCoFeCr samples after irradiation with He^2+^ ions revealed small (less than 10%) changes in the initial composition, as can be seen in Table 1. In turn, for the NiCoFeCrMn system, changes in the concentration of elements are 15–17%. The revealed changes in the concentration of elements in the irradiated samples can be associated with the accumulation and diffusion of mobile helium atoms, as well as the features of the HIRBS/PIXE technique. However, the concentration of elements in the irradiated samples remains close to equiatomic and also uniform over the entire analyzed depth (Figure 2), which indicates the resistance of these alloys to the occurrence of concentration gradients, i.e., the radiation-stimulated segregation.

Microscopic studies of the surface of the HEA after irradiation with helium ions with a maximal fluence (2 × 10^17^ cm^−2^) did not reveal changes in the surface morphology of the samples (Figure 3e,f) and traces of erosion. As can be seen in the figures, irradiation with helium ions does not lead to a change in the grain structure of a HEA. According to the literature [51], blister formation in materials occurs when a critical fluence is reached. For pure metals, the critical dose is about (2–3) × 10^17^ cm^−2^. In our case, the formation of blisters in Ni samples was found at a fluence of 2 × 10^17^ cm^−2^ (Figure 3d). The blisters were 7–11 microns in diameter. At the same time, at a fluence of 5 × 10^16^ cm^−2^, the formation of blisters in nickel samples was not detected. This confirms the high resistance of the microstructure of the HEA surface to irradiation with helium ions.

The X-ray diffraction analysis (SAXRD) of Ni and alloy samples irradiated with He^2+^ ions was carried out for X-ray penetration depths of up to 77 nm (Region 1) and 77–231 nm (Region 2). According to Figure 8, Region 1 corresponds to a low radiation damage and concentration of implanted helium (compared to the maximum), and Region 2 includes the maximum concentration of implanted helium and maximal damage dose (23 dpa).

The analysis of X-ray diffraction patterns of samples after irradiation with helium ions (Figure 4) did not reveal the appearance of diffraction peaks corresponding to new phases or the disappearance of existing ones, i.e., there was no decomposition of solid solutions for NiCoFeCr and NiCoFeCrMn alloys. This indicates a high radiation resistance of the HEA phase composition to irradiation with helium ions with a fluence of up to 2 × 10^17^ cm^−2^.

A shift of the diffraction peaks of solid solutions to the region of smaller angles (Figure 4b,c) for a fluence of 5 × 10^16^ cm^−2^ was found, which indicates an increase in the lattice parameter of the solid solution, which is associated with the processes of defect formation during irradiation with helium ions. The relative change in the lattice parameter of the (Ni, Co, Fe, Cr) solid solution calculated from the X-ray diffraction patterns was 0.047%, and for (Ni, Co, Fe, Cr, Mn), it was 0.088%. An increase in the lattice parameter of solid solutions with an increase in fluence from 5 × 10^16^ to 2 × 10^17^ cm^−2^ by 0.16% and 0.24% was also revealed for NiCoFeCr and NiCoFeCrMn HEAs, respectively. An increase in the lattice parameter of solid solutions is associated with the accumulation of radiation defects and an increase in the density of helium vacancy clusters. At the same time, it should be noted that for the NiCoFeCrMn alloy, a larger increase in the lattice parameter of the solid solution was revealed compared to NiCoFeCr, which is consistent with the data obtained by Y. Tong et al. [40]. For the Ni sample, a decrease in the lattice parameter by 0.15% was revealed after irradiation with a fluence of 2 × 10^17^ cm^−2^.

Analysis in Figure 4 showed that for X-ray patterns obtained at α = 1.19–1.20° (Region 2) and a fluence of 2 × 10^17^ cm^−2^, an asymmetry (shoulder) of the diffraction peaks of solid solutions from the side of the smaller 2θ (marked in Figure 4 with an asterisk (*)) exists. At the same time, a comparison with the X-ray pattern obtained at α = 0.39° revealed that the asymmetric diffraction peaks are a superposition of the two peaks, one of which is close to the diffraction peaks for α = 0.39°. This indicates that the shoulder of the asymmetric diffraction peak is related to the reflection from the phase of the strongly deformed solid solution in Region 2 (the maximal damage dose and concentration of implanted helium). For Ni, the formation of an asymmetric peak shoulder was also found (Figure 4a). A comparison of Ni diffraction spectra obtained for different angles α showed that the formation of an asymmetric peak shoulder is associated with diffraction from Regions 1 and 2 (Figure 8). In view of the small angle of incidence of the X-ray beam (0.084°), as well as the formation of blisters on the nickel surface, it is impossible to accurately determine the depth of X-ray penetration into Ni for a given α.

To assess the possible change in internal stresses after irradiation, studies of macrostresses in samples were carried out using the g-sin^2^ψ method. The results of determining the residual stresses in the unirradiated and irradiated samples at α = 0.084 and 0.39° (Region 1) and 0.25 and 1.19, 1.20° (Region 2), calculated for the (111) orientation, are shown in Figure 5. As can be seen in Figure 5, irradiation of HEAs with helium ions leads to a change in stresses from tensile (unirradiated) to compressive (irradiated). Already at a fluence of 5 × 10^16^ cm^−2^, the level of compressive microstresses is 100–150 MPa. With an increase in the fluence of helium ions from 5 × 10^16^ cm^−2^ to 2 × 10^17^ cm^−2^, the level of compressive stresses increases. As seen in Figure 5, the maximal macrostresses in Region 1 are −268 MPa, and in Region 2, they are equal to −657 MPa. Irradiation of nickel leads to a decrease in tensile stresses, and at a fluence of 2 × 10^17^ cm^−2^, to the formation of a low level of compressive stresses of about 30–50 MPa. Figure 5 shows that at a fluence of 5 × 10^16^ cm^−2^, the stress levels in Regions 1 and 2 are close, and at a fluence of 2 × 10^17^ cm^−2^, the stresses in Region 2 are 2.2–2.5 times higher. This indicates that, in the region of the maximal damage dose and helium concentration, the density of radiation defects, dislocations and helium vacancy clusters sharply increases with increasing fluence, which is associated with an increase in the concentration of the implanted helium and damage dose.

An important point is the comparison of the behavior of macrostresses in the HEAs of NiCoFeCr and NiCoFeCrMn. As seen in Figure 5, the stress level in NiCoFeCrMn alloys is higher and they grow faster with increasing fluence than in NiCoFeCr. This indicates a high density and low mobility of radiation defects (especially helium vacancy clusters) in the NiCoFeCrMn HEA. It is known that the HEAs of NiCoFeCrMn after irradiation are characterized by a lower radiation swelling and damage, the absence of radiation-induced segregation, a higher density of dislocation loops and distortion of the solid solution lattice compared to the HEAs of NiCoFeCr [40,52,53,54,55,56].

The analysis of microstresses and dislocation density of irradiated samples (Figure 6 and Figure 7) showed that irradiation of HEA with helium ions leads to an increase in the level of compressive microstresses. An increase in fluence also leads to an increase in the level of compressive microstresses. The behavior of microstresses in irradiated HEAs correlates with changes in macrostresses. In Region 1, the microstresses in the HEAs of NiCoFeCr and NiCoFeCrMn coincide within the error, and in Region 2, they increase significantly. At the same time, the level of microstresses in the NiCoFeCrMn HEA is higher than in NiCoFeCr, which is in good agreement with the data of macrostresses (Figure 5). It should also be noted that the level of microstresses in the Ni samples is lower compared to HEAs upon irradiation with helium ions with a fluence of 5 × 10^16^ cm^−2^, which may be due to the formation of larger helium vacancy clusters and their low density [57]. A further increase in fluence leads to the formation of helium bubbles, their growth and the formation of blisters in Ni (Figure 3d).

Irradiation with helium ions and an increase in the ion fluence also lead to an increase in the dislocation density (Figure 7). For Region 1, irradiation with helium ions leads to an increase in the dislocation density by a factor of 2–3 for a fluence of 5 × 10^16^ cm^−2^ and by a factor of 4–5 for a fluence of 2 × 10^17^ cm^−2^ (Figure 7a). At the same time, for Region 2, the dislocation density increases 5–12 times for a fluence of 5 × 10^16^ cm^−2^ and 30–60 for 2 × 10^17^ cm^−2^ (Figure 7b). The data of macro–microstresses and dislocation density indicate a significant increase in the density of radiation defects in the HEA. The dislocation density in the Ni samples varies in the same way as in the HEA, while the values are 2–3 times lower. It was shown in [58] that irradiation of HEA NiCoFeCr ions with 3 MeV Ni at 580 °C to 5 × 10^16^ cm^−2^ leads to an increase in the dislocation density in the region of maximal damage by a factor of 5. The dislocation density increases with depth with an increasing damage dose and then sharply decreases in the region of the maximal concentration of the implanted helium. The dislocation density in HEAs depends on the amount of point defects and their mobility in three dimensions, which in turn depend on helium. It was shown that dislocations can form from the agglomeration of point defects, and the dislocation density increases with irradiation fluence for NiCoCrFePd HEA irradiated by Xe^3+^ ions [59]. Helium irradiation also affects the diffusion of point defects, and therefore changes the distribution of the dislocations in HEAs [31]. Therefore, the higher increase in dislocation density in the maximal implantation region can be associated with the accumulation of helium and formation of low-mobility defects there.

The obtained data on the change in macro–microstresses and the dislocation density testify to the high radiation resistance of HEAs compared to nickel. The analysis of these data also makes it possible to compare the radiation resistance of NiCoFeCr and NiCoFeCrMn HEAs.

## 4. Discussion

The elemental, phase composition and microstructure of the surface of the considered HEAs, NiCoFeCr and NiCoFeCrMn, are resistant to irradiation with helium ions (40 keV, 2 × 10^17^ cm^−2^), as no formation of new phases or surface erosion was found.

Thus, the main changes resulting from the irradiation of the samples are associated with the formation and interaction of radiation defects, as well as the formation and redistribution of stresses in alloys.

Irradiation with helium ions led to lattice shrinking in Ni and lattice expansion in NiCoFeCr and NiCoFeCrMn. The increase in the lattice parameter can be caused by the microstrain and defects generated by helium irradiation [60], where point defects and small defect clusters increase the strain and lattice parameter, while larger clusters, such as dislocation loops, can reduce them [40]. The lattice expansion and microstrain decrease at higher irradiation temperatures, since it stimulates defect recombination, reduces the residual stress and lattice distortion, and the expansion depends not only on the irradiation conditions, but also on the constituent elements of the target [39,41]. The addition of Fe and Mn reduce the volume swelling in the Ni alloys [53]. In this study, the lattice expansion in FCC NiCoFeCrMn samples irradiated by helium ions to a dose of 23 dpa was approximately 0.35% relative to unirradiated samples, which was lower than in FCC high-entropy ceramics (WTiVNbTa)C_5_ irradiated by carbon ions to comparable a dose of 23 dpa at room temperature [41], and lower than in BCC HEA VCrFeMn irradiated by He to 1.3 dpa at 1023 K [39]. Therefore, the difference in the lattice expansion between Ni, NiCoFeCr and NiCoFeCrMn is associated with the size of the defects, dislocation density, lattice distortion and elemental composition.

It is known that helium bubbles in NiCoFeCrMn HEAs have a smaller size, higher density, denser distribution and a lower volume fraction compared to NiCoFeCr HEAs and nickel [29,61]. This indicates that NiCoFeCrMn alloys have a stronger resistance to He bubble formation. Typically, helium accumulation and bubble formation in metals is controlled by helium diffusion, which is influenced by irradiation conditions such as temperature, damage rate and the helium formation rate [62,63].

When irradiated with helium ions, the formation of He is always accompanied by the creation of many paired Frenkel defects (vacancy and interstitial) [50]. Since the solubility of He in a metal system is limited [62], the implanted He atoms will diffuse and bond with vacancies to form bubbles, and the behavior of point defects, including their mobility and concentration, will have a significant effect on the behavior of He, as well as on the formation of bubbles. It was assumed in [28] that the chemical disorder of the HEA will lead to the distribution of energy barriers for point defects, and the overlap of the migration energies of interstitials and vacancies will promote the recombination of defects in the HEA. This means that the mutual recombination of point defects will be locally enhanced in the HEA of NiCoFeCrMn, so that the concentration of vacancies in it, caused by displacement damage, will be lower than in NiCoFeCr and Ni.

From the atomistic point of view (density functional method), a higher energy barrier is assumed for helium migration into the HEA by insertion or substitution [63], i.e., suppressed helium mobility (the “sluggish diffusion” effect) with an increase in the complexity of the HEA composition [64]. In our case, the He bubbles in Ni are distributed over a wider area when compared under the same irradiation conditions, which means that He can quickly move away from the peak area and contribute to the formation of He bubbles there. The suppressed mobility of helium explains the high level of stresses and dislocation density in the HEA NiCoFeCrMn, which is associated with an increased concentration of helium and a limited volume of its distribution. The decrease in stresses and dislocation density in the HEA of NiCoFeCr, and especially in Ni, is associated with the distribution of helium over a larger volume and the formation of a lower density of helium vacancy clusters.

Thus, an analysis of the behavior of stresses and dislocation density after irradiation makes it possible to compare the radiation resistance of HEAs.

## 5. Conclusions

Bulk alloys based on single-phase solid solutions (Ni, Co, Fe, Cr) and (Ni, Co, Fe, Cr, Mn) with an FCC lattice, a coarse-grained structure (80–100 µm) and a uniform distribution of elements over depth were manufactured. In NiCoFeCr and NiCoFeCrMn alloys, tensile macro- (103 ± 10 and 44 ± 5 MPa) and microstresses (1.05 ± 0.15 and 0.88 ± 0.15 GPa) were revealed, the emergence of which is associated with the mechanical processing of materials at the manufacturing stage.

It was found that the irradiation of NiCoFeCr and NiCoFeCrMn HEAs by He^2+^ ions with the energy of 40 keV at a fluence of up to 2 × 10^17^ cm^−2^ does not lead to a change in the elemental and phase composition, as well as to erosion of the sample surface. It was revealed that irradiation of NiCoFeCr and NiCoFeCrMn HEAs with helium ions with a fluence of 5 × 10^16^ cm^−2^ leads to the formation of compressive stresses (−90 ... −160 MPa) and their growth over −650 MPa with an increase in fluence of up to 2×10^17^ cm^−2^. The greatest increase in the stress level was revealed for the region of the maximal damage dose and helium implantation.

Irradiation with He^2+^ ions also leads to an increase in the level of compressive microstresses of up to 2.7 GPa at a fluence of 5 × 10^16^ cm^−2^, and up to 6.8 at 2 × 10^17^ cm^−2^. An increase in the dislocation density by a factor of 5–12 for a fluence of 5 × 10^16^ cm^−2^, and by 30–60 for a fluence of 2 × 10^17^ cm^−2^ was also revealed. The greatest changes in stresses and dislocation density are established for the region of maximum damage dose and concentration of implanted helium.

It has been determined that the HEAs of NiCoFeCrMn are characterized by a higher level of macro- and microstresses, dislocation density, as well as a larger increase in their values with increasing helium ion fluence compared to NiCoFeCr.

It has been established that NiCoFeCrMn HEAs are characterized by a higher radiation resistance compared to the NiCoFeCr alloy.

## Figures and Tables

**Table 1:** Elemental composition of CoCrFeNi and CoCrFeMnNi unirradiated and irradiated by He^2+^ ions (40 keV, 5 × 10^16^ cm^−2^ and 2 × 10^17^ cm^−2^).

Sample	Concentration of Elements, at.%				
	Ni	Co	Fe	Cr	Mn
CoCrFeNi (unirradiated)	17.9	28.9	26.2	26	–
CoCrFeNi (Не^2+^, 5 × 10^16^ cm^−2^)	19.8	27.5	27.7	25	–
CoCrFeNi (Не^2+^, 2 × 10^17^ cm^−2^)	18.5	31	25	25.5	–
CoCrFeMnNi (unirradiated)	17.9	22.3	17.9	20	21.9
CoCrFeMnNi (Не^2+^, 5 × 10^16^ cm^−2^)	19.6	18.8	18.3	21.3	22
CoCrFeMnNi (Не^2+^, 2 × 10^17^ cm^−2^)	21	19.7	20.6	18.4	20.3

## Data Availability

The authors declare that the data supporting this study are available from the corresponding author upon request.
