# Correction: Amanzhulov et al. Composition and Structure of NiCoFeCr and NiCoFeCrMn High-Entropy Alloys Irradiated by Helium Ions. *Materials* 2023, *16*, 3695

**DOI:** 10.3390/ma17246290

**Published:** 2024-12-23

**Authors:** Bauyrzhan Amanzhulov, Igor Ivanov, Vladimir Uglov, Sergey Zlotski, Azamat Ryskulov, Alisher Kurakhmedov, Mikhail Koloberdin, Maxim Zdorovets

**Affiliations:** 1 Institute of Nuclear Physics, Almaty 050032, Kazakhstan; igor.ivanov.inp@gmail.com (I.I.); ryskulov_nbd@mail.ru (A.R.); kurahmedov.alisher@gmail.com (A.K.); koloberdin@inp.kz (M.K.); mzdorovets@inp.kz (M.Z.); 2 Department of Nuclear Physics, New Materials and Technologies, Physical-Technical Faculty, L.N. Gumilyov Eurasian National University, Astana 010008, Kazakhstan; 3 Engineering Profile Laboratory, L.N. Gumilyov Eurasian National University, Astana 010008, Kazakhstan; 4 Department of Solid State Physics, Belarusian State University, 220030 Minsk, Belarus; uglov@bsu.by (V.U.); zlotski@bsu.by (S.Z.)

In the original publication [1], there were mistakes in Figure 2 and the legend for Figure 2 as published. Figure 2c is a duplicate of Figure 2b. This happened due to a technical error when collecting all six RBS spectra into one Figure 2. “HIRBS/PIXE” in the legend for Figure 2 should be updated to “HIRBS”. The reason is that the combination of HIRBS/PIXE methods was used for calculating Table 1 concentrations, but the spectra in Figure 2 are HIRBS (Heavy Ion RBS) spectra. The corrected Figure 2 and legend appear below.

Accordingly, the reference to “HIRBS/PIXE” has been changed to “HIRBS” in Section 3.1. Composition and Structure of Unirradiated HEAs CoCrFeNi and CoCrFeMnNi, paragraph 3.

Since Bauyrzhan Amanzhulov, Sergey Zlotski and Maxim Zdorovets’s original email addresses are no longer in use, they have been updated to “amanzholovb96@gmail.com”, “zlotski@bsu.by” and “mzdorovets@inp.kz” respectively.

The authors state that the scientific conclusions are unaffected. This correction was approved by the Academic Editor. The original publication has also been updated.

## Figures and Tables

**Figure 2:**
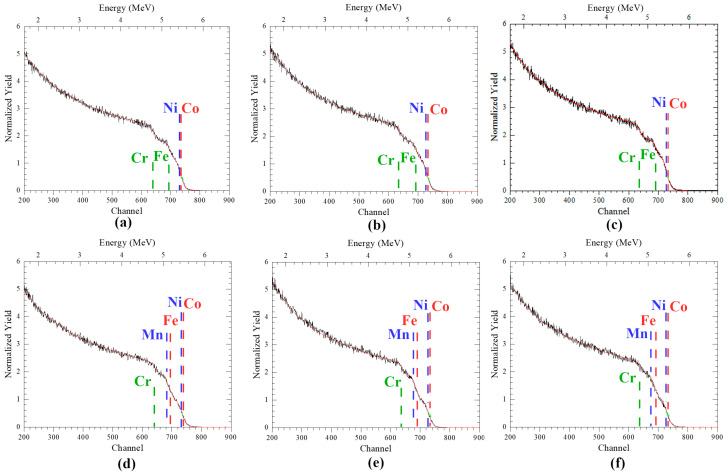
HIRBS spectra of: (**a**,**d**) unirradiated and irradiated by He^2+^ ions (40 keV) with fluences of (**b**,**e**) 5 × 10^16^ cm^−2^ and (**c**,**f**) 2 × 10^17^ cm^−2^, (**a**–**c**) CoCrFeNi and (**d**–**f**) CoCrFeMnNi HEAs.
